# Antioxidant Activity, Glycemic Response, and Functional Properties of Rice Cooked with Red Palm Oil

**DOI:** 10.1155/2024/3483292

**Published:** 2024-05-02

**Authors:** Samsu U. Nurdin, Siti Nurdjanah, Ramadhan Triyandi, Bambang Nurhadi

**Affiliations:** ^1^Department of Agriculture Product Technology, Agriculture Faculty, Lampung University, Bandar Lampung 6235145, Indonesia; ^2^Department of Pharmacy, Faculty of Medicine, Lampung University, Bandar Lampung 6235145, Indonesia; ^3^Department of Food Technology, Agriculture Technology Faculty, Padjadjaran University, Bandung 62 45363, Indonesia

## Abstract

High rice consumption levels accompanied by a lifestyle lacking in physical activity leads to obesity and diabetes due to the rice consumed generally has high digestibility and high glycemic index. Red palm oil (RPO) is a vegetable oil suggested to have the potential to reduce starch digestibility and increase the bioactive compounds of rice. This research aimed to find out the best cooking method to produce rice with a sensory quality similar to regular rice and to study the effect of the best cooking method on the glycemic response and physicochemical properties of rice. The results showed that RPO addition increased the antioxidant activities and total carotenoid levels of rice. The addition of RPO after cooking has better antioxidant activity and total carotenoid than before cooking. Adding 2% RPO before or after cooking produced rice with similar or better sensory quality than regular rice. Rice cooked with 2% RPO added before cooking had a lower glycemic response than regular rice, which was suggested to be caused by the increasing formation of the amylose lipid complex and the triglycerides that protected the starch from amylase enzyme. The formation of the amylose lipid complex and triglyceride layers protecting rice starch was confirmed by the new peaks of the FTIR spectra, the appearance of oil-coated starch morphology, and the changes in the proportion of C and O atoms. In conclusion, the addition of 2% RPO before the cooking process can be considered as a cooking method to produce rice for diabetic patients.

## 1. Introduction

Rice is the staple food of Indonesian people and an essential energy source. High rice consumption levels accompanied by a lifestyle lacking in physical activity leads to obesity and insulin resistance [1] due to the rice consumed generally has high digestibility and high glycemic index. Reducing starch digestibility can be chosen as a strategy to reduce the glycemic index of rice, so the consumption of the rice is not considered a risk factor for type 2 diabetes mellitus (DMT2) [2]. The rice starch digestibility can be reduced by converting the starch to be resistant to digestive enzymes. One of the processes that can convert starch into resistant starch is adding fatty acids or edible oil during processing to form an amylose-lipid complex [3] or type 5 resistant starch (RS5).

Some studies have proven that resistant starch consumption may help prevent DMT2. Consumption of resistant starch can reduce postprandial glucose levels and insulin incremental area under the curve (iAUC) of diabetes patients [4]. Diabetic mice fed a high-fat diet treated with resistant starch showed a dramatic reduction in fasting blood sugar, triglyceride, and total cholesterol levels and were able to increase insulin sensitivity [5]. Giving resistant starch to obese patients with DMT2 reduces fasting glucose levels and insulin concentration [6].

Red palm oil (RPO) is palm oil with a low purity level rich in bioactive compounds such as carotenoid, tocopherols, and tocotrienols [7] with abundant availability. Carotenoids have prominent role in preventing DMT2, primarily through their role as antioxidants [8]. Therefore, it is suggested that the addition of RPO to the rice cooking process will reduce the glycemic index of rice and increase the concentration of rice's bioactive components, especially the carotenoid.

The formation of the starch lipid complex is influenced by various factors, such as the type of fat used [9], the ratio of amylose and lipid, the type of starch [10], and the steps of the starch gelatinization process [11]. In this study, the starch was in the food matrix; therefore, it was suggested that its reaction with lipid would be different from pure starch. Previous studies generally used pure fatty acids or triglycerides [9, 10, 12] as a lipid source for producing RS5. In our study, the triglycerides used contained various bioactive compounds, presumably influencing the formation of resistant starch. Therefore, this study aimed to determine the best concentration of RPO that could be added and the time when the RPO should be added during cooking process that could produce rice with a low glycemic response, high antioxidant activity, and high carotenoid levels that is also acceptable as a staple food.

## 2. Materials and Methods

### 2.1. Materials

The rice used in this study was medium grain rice (IR 64 variety), while the RPO was a commercial red palm oil (Salmira, Indonesia). Chemicals for analysis, such as 1,1-diphenyl-2-picrylhydrazyl (DPPH), ABTS, and *β*-carotene, were purchased from Sigma or other companies with analytical-grade quality.

## 3. Methodology

This research has been approved by the Research Ethical Commission, Faculty of Medicine, University of Lampung (No. 544/UN26.18/PP.05.02.00/2023). This research consisted of two steps. The first step aimed to study the effect of RPO concentration on the antioxidant activity and total carotenoid content of rice and to find out the best cooking method for producing rice with a sensory quality similar to or better than regular rice (RR). The second step aimed to study the effect of the best cooking method on the glycemic response, FTIR spectrum, morphology, and pasting properties of rice. The first study was arranged in a factorial randomized complete block design with the first factor being the concentration of RPO added to the rice (0%, 1%, 2%, 3%, and 4% (w/w)) and the second factor being the time of RPO addition (before and after the cooking process). The treatment in the second step was determined after getting the best treatment from the first step and arranged in a completely randomized design. The data were analyzed with analysis of variance to determine whether there was an effect between treatments. For postdata analysis, the least significant difference test (LSD) was applied at the 5% significance. The data analysis was performed using the type 24 SPPS.

### 3.1. Making Red Palm Oil Rice Flour

The RPO rice flour was prepared by weighing 200 grams of raw rice and then washing it thoroughly under running water. The washed rice was placed in the rice cooker (Miyako), and 400 ml of water was added. The RPO (Salmira) was added according to the treatment: after the rice was cooked or before the rice cooking process. Then, the cooked rice with added RPO was dried in a 50°C oven until dry and ground using a grinder to produce RPO rice powder for further analysis.

#### 3.1.1. Free Radical Scavenging Activity Test Using the DPPH Method

The radical scavenging activity of the RPO rice was determined using the 1,1-diphenyl-2-picrylhydrazyl (DPPH) method according to the previous method [13] with slight modifications. DPPH is one of the antioxidant analytical methods that the most common and widely applied in food and pharmaceutical applications [14]. 7.8 mg of DPPH was dissolved in 100 mL of 96% ethanol to make the stock solution. In a test tube, 100 *µ*l of rice hexane extracts (or hexane as a control) were mixed with 1 mL of DPPH stock solutions and 3 mL of ethanol, and then the tubes were kept in complete darkness for 30 min at room temperature. The absorbance was therefore determined at 517 nm (Inesa, 722G). The following formula was used to calculate the percentage of antioxidant activity:

(1) $$\begin{array}{c} \%\text{ of antioxidant activity}=\left[\left(\text{Ac}-\text{As}\right)÷\text{Ac}\right]\times 100 \end{array}$$

where Ac = Control; As = Testing absorbance.

#### 3.1.2. Free Radical Scavenging Activity Test Using the ABTS Method

The ABTS method measures sequential hydrophilic and lipophilic antioxidant activity; therefore, the obtained antioxidant activity values can be considered as the total of both types of antioxidants [15]. Testing the antioxidant activity with the ABTS method referred to in the research by [13]. The ABTS radical stock solution was prepared by mixing 7 mm of ABTS in ethanol and 2.45 mM of potassium persulfate (1 : 1) then incubating it in a dark at room temperature for 16 h before use. The stock solution was then diluted with ethanol to get an absorbance of 0.700 at 734 nm (considered the control absorbance, AC). Antioxidant activity was measured by mixing 100 *µ*L of sample extract with 2.9 mL of diluted ABTS radical solution, then, after 30 min, measuring the absorbance at 734 nm (Inesa, 722G) (considered as sample absorbance, As). The antioxidant activity was calculated using the formula: Antioxidant activity (%) = ((AC − As)/AC) × 100 (2), where AC is control absorbance and As is sample absorbance.

#### 3.1.3. Analysis of Total Carotenoid Levels

The analysis of total carotenoid levels was carried out following the previous method [16]. Approximately 0.5 g of rice powder was added to a tube containing 2 ml of the ethanol/hexane (1 : 1) mixture, then shook for 10 min at 100 rpm. After centrifugation, the supernatant was transferred to another tube, and the absorbance was read at 446 nm (Inesa, 722G). For the total carotenoid determination, *β*-carotene was used to make the standard calibration curve. A stock *β*-carotene solution was prepared by dissolving 10.0 mg of *β*-carotene in 10.0 mL of the ethanol/hexane mixture. Then, the standard solutions of *β*-carotene were prepared through serial dilutions using the mixture of ethanol/hexane (5–20 *μ*g/mL). The concentration of total carotenoid content in the test samples was calculated from the calibration plot and expressed as ppm *β*-carotene equivalent (BCE) of dried rice.

#### 3.1.4. Sensory Quality Test

The sensory quality test used the focus group discussion (FGD) method, referring to research by Rodrigues et al. [17] with some modifications. The FGD involved a moderator (the researcher) and panelists who had been interviewed directly and were non-smokers willing to become panelists and eat rice.

The FGD test consisted of two steps. First, the selected panelists were asked to determine the level of preference for the sample being tested by giving a value of 3 if the rice had the same level of preference as regular rice, a value of more than 3 if the rice had a higher level of preference than regular rice, and a value of less than 3 if it had a lower preference level than regular rice. They were also asked to write down their reasonings. Then, the panelists' responses were tabulated and used as a topic of discussion in the second step.

In the second step, led by the moderator, the panelists discussed the results of the first step to select the best treatment and identify the attributes that supported their decision.

#### 3.1.5. Glycemic Response (GR)

Determining the glycemic response involved eight respondents who were healthy, nondiabetic, nonsmokers, had normal fasting glucose levels (60–80 mg/dl), had average body mass index (BMI) values in the range of 18.5–22.9 (kg/m^2^), and aged between 20 to 50 years. The rice's GR measurement referred to the El method [18] that was modified by Nurdjanah et al. [19]. The blood glucose concentration was measured using a blood glucose tester (GlukoDr meter) by taking a drop of capillary blood sample using a lancet. Preceding the GR test, respondents were asked to have adequate rest and overnight fasting for at least 10–12 hours (from 20.00 to 08.00) except for drinking water. Blood samples were taken at 0 minutes (before respondents were given the rice sample (equivalent to 40 g available carbohydrate), and when they were still fasting) and after respondents consumed the rice sample (equivalent to 40 g of carbohydrates), at the 30th, 60th, 90th, and 120th minute. During the test, respondents were asked to relax by sitting in an air-conditioned room. The interval of GR test between types of rice was 4–7 days. The types of rice were regular rice (0% RPO addition), rice with 2% RPO addition before cooking, and rice with 2% RPO addition after cooking. The blood glucose concentrations of respondents were then spread out on two axes, the *x*-axis (as the time in minutes) and the *y*-axis (as the blood glucose concentration), and then the area under the curve (AUC) was calculated.

#### 3.1.6. Rice Fourier Transform Infrared (FT-IR) Spectrophotometer Analysis

The FTIR spectrophotometer (Agilent Cary 630) was used to determine the structure of the samples, referring to the published method with some modifications [20] at the 400/cm to 4500/cm wavenumber with the spectra recorded with a 4/cm resolution. The rice flour was mixed well with potassium bromide (KBr) before the measurement.

#### 3.1.7. Scanning Electron Microscope (SEM) Analysis

Analysis of the starch structure was carried out using SEM (ZEISS EVO MA10). The rice flour sample to be analyzed was placed on the sample holder attached to the carbon tape, and the remaining sample that was not attached was cleaned from the carbon tape. The sample holder was inserted into the SEM sample holder. The SEM-EDX tool had two monitors. The picture was obtained from a sample surface image on SEM, and a graph or diagram on the EDX showed the percentage of elements from the analyzed sample [21].

### 3.2. Pasting Property Analysis with Rapid Visco Analyzer

The pasting properties of rice were analyzed using a Rapid Visco Analyzer-TechMaster (Perten Instruments) following the published method [22]. Three grams of RPO rice flour sample were dissolved in 25 ml of distilled water and put into the RVA tube for analysis, which was carried out for 13 minutes. During the analysis process, the solution was stirred for 10 seconds at 160 rpm. Samples entered by the RVA device were then equilibrated until the temperature reached 50°C for 1 minute. Then, it was heated to 95°C for 3.7 minutes. The heating process was maintained at 95°C for 2.5 minutes. Finally, it was cooled until the temperature dropped to 50°C for 2 minutes.

The results were obtained in the form of peak viscosity (PV), holding viscosity (HV), final viscosity (FV), breakdown value (BD = PV − HV), setback value (SB = FV − HV), and pasting temperature (PT) on the RVA curve.

## 4. Results and Discussion

### 4.1. Effect of RPO Concentration and Addition Timing on the Radical Scavenging Activities and Carotenoid Content

Table 1 shows the ability of the rice extract with added RPO to scavenge DPPH and ABTS radicals. Various compounds contained in rice and RPO can neutralize free radicals, such as phenolic compounds, carotenoids, tocopherols, and tocotrienols [7, 23, 24]. Increasing RPO concentration added to rice before or after cooking increased the DPPH and ABTS scavenging activity, presumably due to the increased antioxidant compounds.

There was no significant difference (*p* < 0.05) between the DPPH radical scavenging activities of rice added with RPO before and after cooking (Table 1). The antioxidant compounds in RPO have different antioxidant activities and heat resistance [25]. Therefore, although the heating process exposed to RPO can reduce the concentration of antioxidant compounds, the reduction does not lessen the extract's ability to neutralize DPPH radicals. The antioxidant activity of an extract depends not only on the concentration of the active compound but also on the type of the compound [26].

Table 1 shows that increasing the RPO concentration added to rice increased the ABTS^*∗*^ scavenging activities, and adding it after cooking produced rice with better antioxidant activity (*p* < 0.05). RPO contains antioxidant compounds such as alpha-carotene, beta-carotene, alpha (*α*)-tocopherol, gamma (*γ*)-tocotrienol, and *γ*-oryzanol which can be degraded due to the cooking process [27]. It is suggested that heating rice during cooking causes destruction of some antioxidant compounds. It was suggested that the destruction of these compounds was detected using the ABTS method but not the DPPH method because the sensitivity of the analytical method to determine antioxidant activity depended on the type of compound being tested, and for measuring the activity of antioxidant compounds that have colors, the ABTS method has better accuracy compared to the DPPH [28].

Rice is a cereal poor in carotenoid content [29]; the addition of RPO increased the total carotenoid content of the rice, and it tended to be higher when the RPO was added after cooking (*p* < 0.05) (Table 1). Carotenoids are the main bioactive compounds in RPO, with the proportion of *β*-carotene that has a good resistance against heating [25, 30] reaching 80% [25]. In cupcakes containing RPO, *α*- and *β*-carotene had 100% retention, while tocopherols and tocotrienols had 95% and 85% retention, respectively [30]. Because the maximum temperature for cooking rice using a rice cooker only reached 100°C [31], it is suggested that carotenoids were not destructed.

Adding RPO to rice after cooking tended to result in higher carotenoid concentration than before cooking, especially for the 3% and 4% RPO concentration (Table 1). Although adding RPO before cooking meant the carotenoids were exposed to heat for longer than when added after cooking, the decrease in the carotenoid concentration was presumably not due to carotenoid degradation. The maximum temperature of rice cooker was 100°C [31]; therefore, the rice cooking process did not destroy carotenoid compounds [25, 30]. The decrease was presumably due to isomerization [32] or decreased extractability [33] of the carotenoids.

### 4.2. Focus Group Discussion

The results showed that the use of 2% RPO added either before or after cooking resulted in rice with acceptability similar to or higher than regular rice for 83.3% of total panelists (see Table S1 in the Supplementary Material for sensory evaluation data). Meanwhile, rice with 3% and 4% RPO added before or after cooking, even though their hedonic score (HS) was statistically not different from regular rice, was considered similar to or better than regular rice by less than 70% of the panel.

RPO addition to increase carotenoid content of food has been carried out by previous researchers with different concentrations depending on the type of food. Sorghum cake containing 20% RPO was preferred over the cake with 24% RPO [34] and beef sausages with 10% RPO were more acceptable than those with 15% RPO [35]. In this study, based on the panel's discussion, it was concluded that adding 2% RPO, either before or after cooking, could be used as a cooking method to produce rice with a sensory quality similar to or better than regular rice. The FGD of panelist concluded that the dislike responses of the panelists were generally caused by the oily taste and aroma, especially for rice with 3% and 4% RPO. Therefore, rice cooked with 2% RPO was further evaluated for its glycemic response and physicochemical properties.

### 4.3. Glycemic Response of Rice

**Figure 1:**
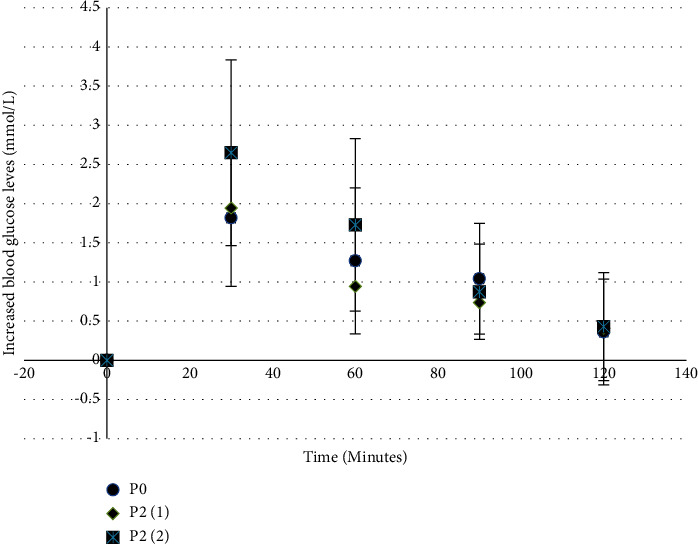
The effect of RPO concentration and addition timing on the respondent's increased blood sugar levels consuming original rice (P(0)) rice added with 2% RPO before (P2(1)) and after (P2(2)) cooking.

Figure 1 shows the effect of the consumption of regular rice (P0) as well as rice added with 2% RPO before (P2(1)) and after (P2(2)) cooking on the respondents' blood sugar levels for 120 minutes. From Figure 1, it can be seen that after consuming rice, the panelists' blood sugar levels reached their peaks at 30 minutes with 120 mg/dl (P0), 117 mg/dl (P2(1)), and 129 mg/dl (P2(2)). These results are in line with previous glycemic response studies on the consumption of rice cooked with spices where respondents also reached their peak blood sugar levels at 30 minutes [36].

**Figure 2:**
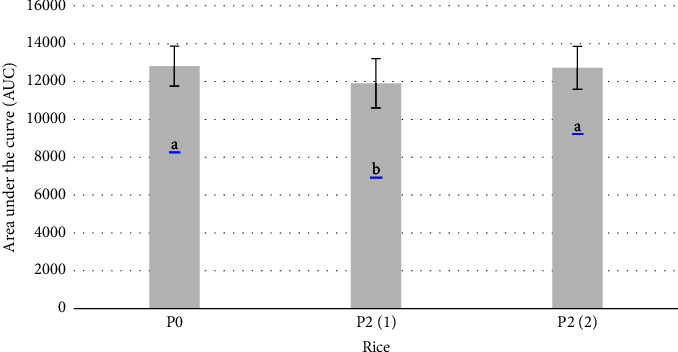
Effect of RPO concentration and addition timing on the area under the curve of regular rice (P(0)), rice added with 2% RPO before (P2(1)) and after (P2(2)) cooking. Data are means ± standard deviations. Data points denoted by different superscripts (letter on the bars) differs significantly with *p* < 0.05.

The area under the curve (AUC) of the respondents' blood levels after consuming rice can be seen in Figure 2, as it represents the glycemic response of the rice samples. Figure 2 shows that rice cooked with 2% RPO added before cooking (P2(1)) has a smaller AUC than regular rice (P0) and rice cooked with 2% added after cooking (P2(2)); therefore, consumption of P2(1) rice will result in a lower increase in blood sugar than that of P0 or P2(2) rice. It was suggested that the addition of 2% RPO before cooking decreased the glycemic response of rice due to a decrease in the starch digestibility of rice.

The decrease of starch digestibility might be caused by the formation of RS5 and triglyceride protection that prevents starch hydrolysis by amylase enzyme [37, 38]. RS5 is formed when amylose forms complexes with fatty acids [39], thus making it resistant to digestion. Triglycerides in RPO presumably form a layer that covers starch; as a result, the starch is protected from enzymes attack [38]. The triglycerides added after cooking were suggested to only coat the surface of the rice grains and did not provide uniform protection due to their hydrophobic properties. Meanwhile, the addition before cooking allowed the triglycerides to be dispersed evenly due to the heating process that increased the solubility of oil in water [40].

### 4.4. FTIR Spectrum of Rice

**Figure 3:**
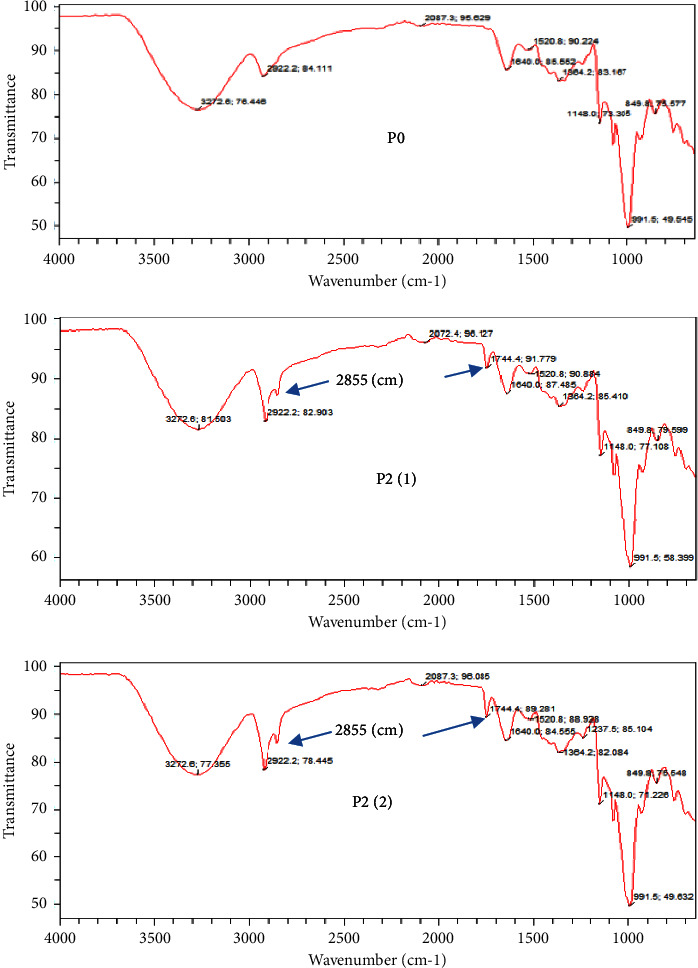
FITR spectrum of regular rice (P0) and rice added with 2% RPO before (P2(1)) and after (P2(2)) cooking.

The FTIR spectrum of regular rice (P0) and rice added with 2% RPO (P2(1) and P2(2)) can be seen in Figure 3. Figure 3 shows that the –OH stretching vibration absorption peak occurred between 3600/cm and 3100/cm [20], and the most intense appeared at 3272/cm. A strong absorption peak appeared in all samples.

In the spectral range of 4,000∼650/cm, the shape and position of infrared absorption peaks of P2(1) and P2(2) were similar compared to P0, except for the new characteristic peaks appearing at 1744/cm, which indicated that the new group was generated after RPO addition. This wavenumber indicates the presence of triglyceride ester group vibrations (C=O) [41] or C=C bonds in fats or fatty acids [42]. The addition of RPO before and after cooking increased the triglyceride and fatty acid levels of rice.

### 4.5. Morphology of Rice Starch

**Figure 4:**
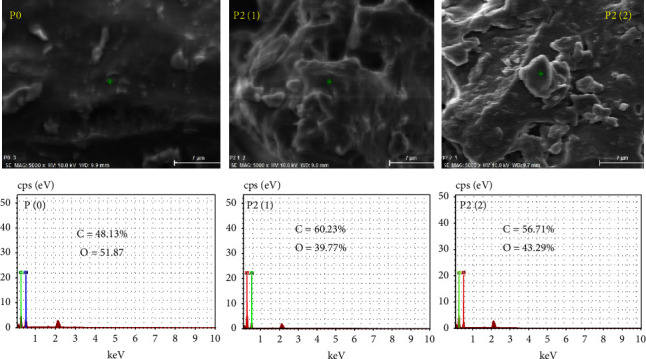
Morphology and proportions of C and O atoms of regular rice (P0) and rice added with 2% RPO before (P2(1)) and after (P2(2)) cooking.

Observation of the morphology of starch granules using SEM was carried out on regular rice (P0) and rice added with 2% RPO before (P2(1)) and after (P2(2)) cooking (Figure 4). The results showed that rice cooked with the addition of 2% RPO (P2(1) and P2(2)) had an oily surface unlike regular rice (P0), and the impression of oiliness in P2(2) was more visible compared to P2(1).

The main composition of RPO is triglycerides that do not form complexes with rice starch [43]. During cooking, the triglycerides in the RPO added before cooking (P2(1)) would be completely dispersed in the water used for cooking, therefore coating the rice granules not only on the surface but also penetrating the inner part when the rice swelled during gelatinization. Meanwhile, the triglyceride in RPO added after cooking (P2(2)) did not have a dispersing medium because the rice had completely absorbed the water; as consequence, the triglycerides only coated the rice surface after mixing, and the rice looked oilier.

The RPO addition also modified the proportion of C and O atoms of rice, from 48.13% C and 51.87% O in regular rice to 60.23% C and 39.77% in P2(1) and 56.71% C and 43.29% O in P2(2) (Figure 4). Depending on the rice variety, the proportion of C in rice can be more or less dominant than O [44]. In this study, the rice used had a lower proportion of C than O. Because the lipid in RPO presumably had a higher proportion of C than O [45], the RPO addition significantly increased the proportion of C in rice.

### 4.6. Pasting Properties of Rice

The rapid visco analyzer (RVA) measures changes in sample viscosity during heating and cooling, which are then interpreted as the pasting properties of the sample.  Table 2 shows that the RPO addition affected the pasting properties of the cooked rice except for the pasting temperature (PT). Previous studies have shown that the formation of amylose lipid complex increases starch's PT due to the inhibited swelling of starch granules which causes a slower gelatinization process [46]. However, in this study, the formation of the amylose lipid complex did not affect PT. It was presumed that the complex formed was not sufficient for altering the pasting temperature of the starch [47].

Peak Viscosity (PV) and Hold Viscosity (HV) decreased after RPO addition (Table 2). Devi et al. [22] reported a decrease in PV due to the addition of vegetable oil, which was suspected to be caused by the swelling inhibition of starch granules by fatty acids. HV measures the viscosity when the expanded starch granules are damaged by pressure and heat [48]. Adding RPO to the rice cooking process is thought to cause the expanded starch granules to be more easily damaged, causing a more significant decrease in viscosity compared to regular rice. The instability of the expanding granules is also supported by the higher breakdown (BD) values of rice added with RPO compared to regular rice (Table 2). BD is the difference between peak viscosity (PV) and hold viscosity (HV), where if the BD value is higher, the stability is lower [49].

FV is generally used to determine the quality of starch flour because it describes the ability of the starch to form a thick paste after heating and cooling. The addition of RPO lowered the FV values (Table 2), which means that the addition of RPO reduced the ability of rice starch to form a thick gel. SB is the difference between FV and HV, indicating the gel paste's hardness after cooling and the degree of ease of retrogradation [48].  Table 2 shows that the addition of RPO lowered the SV values, so rice added with RPO tended to be more difficult to experience retrogradation. This was probably due to a decrease in the ability to form amylose-lipid complexes, with added RPO [50].

## 5. Conclusion

The study showed that adding RPO to rice increased its antioxidant activity and total carotenoid levels of rice. Cooking rice with the addition of 2% RPO before or after cooking produced rice with sensory quality that was similar to or better than regular rice. Rice cooked with 2% RPO added before cooking had a lower glycemic response than regular rice, presumably caused by the increased formation of the amylose lipid complex or by triglycerides that protected starch from amylase enzyme attack. The formation of amylose lipid complex and triglyceride layers that protected rice starch was confirmed by new peaks in the FTIR spectra—which indicated the presence of lipid—as well as the appearance of oil-coated starch morphology and changes in the proportion of C and O atoms due to increased lipid concentration. The addition of RPO affected the pasting properties of rice but did not affect the pasting temperature. Therefore, the addition of 2% RPO before cooking can be used as a method of cooking rice for people with diabetes because the rice has a lower glycemic response, higher antioxidant activity and carotenoid content than regular rice, and acceptable sensory quality as a staple food.

## Figures and Tables

**Table 1:** Effect of RPO concentration and addition timing on the DPPH and ABTS radical scavenging activity and total carotenoid content.

Parameters	Addition timing	Red palm oil concentration				
		0%	1%	2%	3%	4%
DPPH radical scavenging activity (%)	Before cooking	1.86 ± 0.7a	3.73 ± 3.1ab	4.28 ± 2.2ab	5.65 ± 1.5b	9.97 ± 5.3c
		f	f	f	f	f
	After cooking	2.12 ± 1.2a	5.39 ± 2.0a	8.17 ± 2.6b	8.32 ± 1.2b	10.40 ± 3.2b
		f	f	f	f	f

ABTS radical scavenging activity (%)	Before cooking	1.81 ± 2.8a	2.75 ± 2.5ab	3.86 ± 2.3ab	5.71 ± 2.8b	10.38 ± 1.9c
		f	f	f	f	g
	After cooking	1.75 ± 0.7a	3.56 ± 0.8a	4.82 ± 2.7a	10.57 ± 2.3b	11.12 ± 1.5b
		f	f	f	g	g

Total carotenoid content (ppm)	Before cooking	94.1 ± 13.8a	136.5 ± 3.9b	187.5 ± 23.1c	246.7 ± 30.0d	278.0 ± 45.5e
		g	g	g	h	h
	After cooking	88.9 ± 4.6	163.1 ± 30.5b	229.9 ± 32.1c	300.6 ± 4.8d	377.0 ± 56.9e
		g	g	g	h	h

Data are means ± standard deviations (*n* = 3). Values within the same row (a, b, c, d, e) and the same column (f, g, h) with different letters are significantly different (*p* < 0.05).

**Table 2:** Effect of RPO concentration and addition timing on the properties of rice paste.

Characteristic	P0(1)	P2(1)	P2(2)
Pasting temperature	94.5 ± 10.6a	85.0 ± 7.1a	96.5 ± 17.7a
Peak viscosity (PV)	2471.0 ± 59.4a	2179.0 ± 41.0b	2098.0 ± 31.1b
Hold viscosity (HV)	2449 ± 49.5a	2120.5 ± 38.9b	2044.0 ± 17.0b
Final viscosity (FV)	4665.5 ± 6.4a	4146.5 ± 109.6b	3936.0 ± 56.6b
Breakdown (BD)	22.0 ± 9.9a	58.5 ± 2.1b	54.0 ± 14.1b
Setback value (SB)	2216.5 ± 55.9a	2026.0 ± 70.7b	1892.0 ± 39.6b

*Note.* Data with the same letter in the row are not significantly different at the 5% significance level.

## Data Availability

The data used to support the findings of this study are available from the corresponding author upon request.

## Supplementary Materials

Supplementary Materials Supplement 1: effect of RPO concentration and addition timing on panelists' preference for rice (*Focus group discussion*).
