# Supplementary material for: Antioxidant Activity, Glycemic Response, and Functional Properties of Rice Cooked with Red Palm Oil
Source: J Nutr Metab. 2024 May 2;2024:3483292. doi: 10.1155/2024/3483292 (PMC11081750; doi:10.1155/2024/3483292)
Supplement: Supplementary Materials — Supplement 1: effect of RPO concentration and addition timing on panelists' preference for rice (Focus group discussion). [file 3483292.f1.docx]

Table S1. Effect of RPO concentration and addition timing on panelists' preference for rice (*Focus group discussion*)

| Organoleptic Parameter | | Addition Timing and Concentration of RPO | | | | | | | |
| --- | --- | --- | --- | --- | --- | --- | --- | --- | --- |
|  |  | Before | | | | After | | | |
|  |  | 1% | 2% | 3% | 4% | 1% | 2% | 3% | 4% |
| Taste | HS | 3.5±0.5 | 3.3±1.0 | 3.2±1.0 | 2.9±1.1 | 3.5±0.7 | 3.5±0.7 | 3.0±0.8 | 3.1±1.0 |
|  | P (%) | 100 | 80 | 60 | 50 | 90 | 90 | 70 | 60 |
| Flavor | HS | 3.0±0.7 | 3.2±0.6 | 3.3±0.7 | 3.8±0.8 | 3.3±0.5 | 3.5±0.7 | 3.1±1.0 | 3.4±1.0 |
|  | P (%) | 80 | 90 | 90 | 100 | 100 | 90 | 60 | 80 |
| Fullness | HS | 3.4±1.0 | 3.4±0.8 | 3.2±1.0 | 2.9±1.0 | 3.2±0.6 | 3.7±0.5 | 3.1±0.9 | 3.3±1.1 |
|  | P (%) | 90 | 90 | 70 | 60 | 90 | 100 | 70 | 70 |
| Mouthfeel | SH | 3.2±0.8 | 3.2±0.8 | 3.0±1.1 | 2.7±1.1 | 3.4±0.8 | 3.2±0.8 | 2.6±0.8 | 2.6±0.8 |
|  | P (%) | 80 | 80 | 60 | 40 | 80 | 40 | 40 | 80 |
| Color | HS | 3.1±1.1 | 3.4±0.8 | 3.4±1.1 | 3.4±1.1 | 2.8±0.8 | 3.5±0.7 | 4.0±0.8 | 3.8±1.0 |
|  | P (%) | 60 | 80 | 70 | 70 | 60 | 90 | 90 | 80 |
| Acceptance | SH | 3.6±0.5 | 3.4±0.8 | 33±1.1 | 3.3±1.0 | 3.6±0.5 | 3.8±0.4 | 3.0±0.7 | 3.2±0.9 |
|  | P (%) | 100 | 80 | 70 | 80 | 100 | 100 | 80 | 80 |
| Average | HS | 3.3 | 3.3 | 3.2 | 3.2 | 3.3 | 3.5 | 3.1 | 3.2 |
|  | P (%) | 85.0 | 83.3 | 70.0 | 66.7 | 86.7 | 85.0 | 68.3 | 75.0 |

Note: HS = hedonic score; P = the proportion of panelists who stated that their preferences for rice added with RPO were the same or they preferred regular rice
